# Sexual reproduction with variable mating systems can resist asexuality in a rock–paper–scissors dynamics

**DOI:** 10.1098/rsos.140383

**Published:** 2015-07-15

**Authors:** Juan Carranza, Vicente Polo

**Affiliations:** 1 Department of Zoology, CRCP Research Center, University of Córdoba, Córdoba 14071, Spain; 2 Biology and Ethology Unit, University of Extremadura, Cáceres 10071, Spain; 3 Departamento de Biología y Geología del Área de Biodiversidad y Conservación, Escuela Superior de Ciencias Experimentales y Tecnología (ESCET), Universidad Rey Juan Carlos, Madrid, Spain

**Keywords:** sex evolution, maintenance of sex, sexual selection, mating systems, twofold cost of sex, rock–paper–scissors

## Abstract

While sex can be advantageous for a lineage in the long term, we still lack an explanation for its maintenance with the twofold cost per generation. Here we model an infinite diploid population where two autosomal loci determine, respectively, the reproductive mode, sexual versus asexual and the mating system, polygynous (costly sex) versus monogamous (assuming equal contribution of parents to offspring, i.e. non-costly sex). We show that alleles for costly sex can spread when non-costly sexual modes buffer the interaction between asexual and costly sexual strategies, even without twofold benefit of recombination with respect to asexuality. The three interacting strategies have intransitive fitness relationships leading to a rock–paper–scissors dynamics, so that alleles for costly sex cannot be eliminated by asexuals in most situations throughout the parameter space. Our results indicate that sexual lineages with variable mating systems can resist the invasion of asexuals and allow for long-term effects to accumulate, thus providing a solution to the persisting theoretical question of why sex was not displaced by asexuality along evolution.

## 1. Introduction

The evolutionary maintenance of sexual reproduction has been considered the main unsolved problem in evolutionary biology [1–4] and it remains challenging after more than 40 years of research. Sexual reproduction may entail costs of different types with respect to asexuality, such as breaking down successful genetic associations ([5,6] see also [7,8]), decoupling male and female fitness [9], expending time and energy to find suitable mates or incurring risks of predation or parasitism associated with mating [10]. However, the major issue in understanding the evolutionary maintenance of sex is how to balance the disadvantages of sexuality in terms of the cost of producing males who provide no resources to offspring, the so-called ‘twofold cost of sex’, which may reduce the reproductive potential of a lineage to one-half per generation ([1–4,11–15]).

The twofold cost of sex entails disadvantages at two levels. One is the competition between species and lineages. Mean fecundity of an asexual lineage may be twofold per generation compared with a sexual one so that, all others being equal, the former may invade the niche of the latter ([2,16] but see [17]). A number of theories have explored how the mean fitness of a sexual population could be higher than that of an asexual one in the long run to compensate the twofold cost of sex. Considerable research effort has been put on the potential benefits of recombination and segregation in hastening the accumulation of beneficial mutations and in the elimination of deleterious mutations [12,14,15,18–26], as well as on the lower ability of asexual lineages to adapt to heterogeneous or rapidly changing environments [4,27–29], or to maintain evolutionary arm races with parasites (reviewed in [30], see also [28]). As a consequence, asexual lineages may have disadvantages in the long term compared with sexual ones (e.g. [18,31–33], but see: [34–38]).

The other, main type of sex disadvantages is at the individual level. Even if asexuals are short-lived, this does not explain why parthenogenetic individuals do not outcompete sexual breeders in the short term as group selection is normally regarded as much weaker than individual selection (e.g. [39,40]). Indeed, much of the literature on the evolution of sex attempts to weigh the short-term advantage of asexual reproduction with the long-term benefits of sex, as well as asking whether sex has significant benefits that already act over shorter time-scales (see e.g. [12,13,17,19,21,24,41–45]). Models show that for the short-term benefits of a sexual female to be twofold they require rather extreme conditions of environmental variation, which are still too extreme even under strong selection from parasites [46–48], when parasites are considered together with the accumulation of deleterious mutations [49–51], or when females are assumed to mate with genetically diverse males [52]. Modifier alleles that produce changes from asexuality to small increases in the frequency of sex may be shown to spread within a small, finite population if genetic drift produces negative linkage disequilibrium, although even the range of population sizes that favours sex are rather small when the twofold cost of sex is included [14,53,54]. Therefore, it seems that in many cases the twofold cost may actually not being compensated in the short term. Hence, the question is still open on how alleles promoting sex are not displaced in the short term by those promoting asexuality [15,55].

The twofold cost of sex originated through competition over mates [4,56,57]. The full twofold cost only occurs when sexual females invest 50% of their resources [11] to produce males that do not contribute with resources to the production of offspring, normally because they invest their entire reproductive budget in competition with other males to secure mates. Therefore, it is polygyny, and the associated male competition for mates, which causes the twofold cost of sex.

Polygynous, costly sex must have been favoured by selection because of their advantages over earlier sexual strategists [56–59]. Alleles that promoted in males the allocation of reproductive effort into mating behaviour rather than into contributing to the production of offspring should have benefited from the higher mating success of their carriers compared with those of less polygynous males. Thus, one evident short-term benefit that might have contributed to compensate for the twofold cost of sex for a sexual female is the likely increment in the ability of her sons to obtain multiple mates. However, as the polygynous system spreads, mean population fitness may have decreased, as well as that of the individual females compared with similar monogamous or parthenogenetic ones, which would have made the population more vulnerable to invasion by asexuals. Thus, our understanding of the maintenance of costly sex should benefit by considering its interactions not only with asexuals but also with low-cost sexuals (when males contribute with variable amounts of resources to offspring production). To our knowledge, no model has so far explored: (i) whether the interactions between several reproductive strategies operating simultaneously in the population may have a role in explaining the maintenance of costly sex, and (ii) especially, whether costly sex may persist under such circumstances without the need of recombination benefits that outweigh the twofold cost.

Our goal in this article is to study the dynamics of alleles that promote costly sex when they coexist with alleles promoting low-cost sexual modes and asexuality, when recombination or genetic segregation cannot offset the full twofold cost of sex. We use game theory in a sexual selection genetic model that investigates the evolutionary dynamics of asexual and sexual individuals that use different reproductive strategies in an infinite population.

## 2. The model

We considered a hypothetical population composed of infinite diploid individuals, without overlapping generations. Individuals may reproduce asexually (parthenogenetically) or sexually. When sexual, they produced even proportions of male and female offspring. The reproductive strategy of individuals was determined by two autosomal two-allele loci with independent segregation. The first locus *S*, influenced whether the reproduction was asexual (allele *a*) or sexual (allele *s*). Genotype *aa* determined an asexual (or parthenogenetic) reproductive strategy *S*_1_; genotype *ss* produced sexual reproduction; and heterozygote *as* individuals may choose between sexual and asexual reproduction with a probability *α* and 1−*α*, respectively. A second locus *M*, influenced the mating system and its associated cost-of-sex, it being non-costly (allele *n*) or costly sex (allele *c*) and only expresses in the presence of allele *s* in locus *S*. Genotype *nn* provided a non-costly sexual strategy *S*_2_, typically due to males contributing as much as females to the resources to offspring (e.g. symmetrical parental care), although it may also encompass negligible expenditure in males or male function in hermaphrodites; and genotype *cc* determined a costly sexual strategy (polygyny) *S*_3_, where males are polygynous and do not provide parental care at all, so that sexual females invest on average half of their resources to produce males that do not contribute to resources to offspring. The behaviour of heterozygotic individuals *nc* depended on the dominance relationship between *n* and *c* alleles indicated by parameter *k* (*k*=1 when allele *c* is dominant, *k*=0 when *c* is recessive, and 0<*k*<1 when *n* and *c* have some degree of codominant relationship).

All females, irrespective of their genotype and the resources provided by males, allocated equal parental resources to offspring. Recombination was assumed to influence fitness, because of differences in any characteristics of sexually produced offspring with respect to asexually produced ones, although we did not deal with the nature of possible recombination benefits. We introduced parameter *R* as the relative benefit of recombination with respect to asexuality. *R* does not include the twofold cost, as the latter is only considered as a reduction in number of offspring.

We used parameter *b*_m_ for the male-relative-to-female potential contribution to offspring production (0≤*b*_m_≤1 for sexuals and *b*_m_=0 for asexuals). Female breeding strategy might not allow symmetrical male contribution (consider, for instance, egg production in many invertebrates or mammalian pregnancy and lactation). Thus, we introduced parameter *b*_f_ as the proportion of potential male care that finally contributes to the production of offspring, let us call it the proportion accepted by the female (0≤*b*_f_≤1), so that the product *b*_m_⋅*b*_f_ is the actual male contribution relative to female (0≤*b*_m_⋅*b*_f_≤1 for sexuals and *b*_m_⋅*b*_f_=0 for asexuals). Table 1 summarizes the parameters used in the model.

**Table 1.** Definition of the parameters and alleles used in the model.

symbol	meaning
*S*	locus for reproductive strategy
*M*	locus for mating system
*a*	allele at locus *S* influencing asexuality
*s*	allele at locus *S* influencing sexuality
*n*	allele at locus *M* influencing non-costly sex
*c*	allele at locus *M* influencing costly sex
*S*_1_	asexual reproductive strategy
*S*_2_	non-costly sexual strategy
*S*_3_	costly sexual strategy
*α*	probability of *as* individual choosing asexual strategy
*k*	dominance effect of allele *c*in locus *M*
*τ*	proportion on sexual individuals in the population
*R*	benefit of recombination with respect to asexuality
*m*	maximum potential number of mates for a polygynous male
1+*η*	mean number of mates mate for polygynous *cc* males in the population
*θ*	mean number of mates of heterozygote *nc* males in the population
1−*π*	mean number of mates of homozygote *nn* males in the population
*b*_m_	male-relative-to-female potential contribution to offspring production
*b*_f_	proportion of male parental contribution accepted by female

We assumed fitness (*W*) of each individual displaying the sexual or asexual strategy being determined by five parameters as follows:

2.1 $$W=\text{rNR}(1+b_{m}b_{f}),$$

*r* being the degree of relatedness from parents to offspring (*r*=0.5 for all sexual strategies and *r* = 1 for asexuals); *N*, the number of descendants produced (we assumed *N*=1 for asexuals and for every sexual individual allocating the whole parental budget to offspring); *R*, the relative benefits of recombination with respect to asexuality; and the product *b*_m_*b*_f_ was the contribution of male-relative-to-female-to-offspring production. In summary, equation (2.1)) yields for sexual (*W*_s_, considering the added contributions of male and female breeders) and asexual (*W*_a_) strategists:

2.2 $$W_{s}=R(1+b_{m}b_{f})$$

and

2.3 $$W_{a}=1.$$

Thus, male contribution to offspring production (in short, parental care), *b*_m_⋅*b*_f_, depended on male and female genotypes (i.e. number of *c* alleles), and on the dominance relationship between *n* and *c* alleles in the *M* locus, as follows. Males with genotype *cc* did not provide parental care at all (*b*_m_=0) and females of the same genotype only accepted a proportion *b* (0≤*b*≤1) of the male care to offspring. Males and females of the genotype *nn* invested equally to offspring and thus *b*_m_=*b*_f_=1. The behaviour of heterozygotic individuals *nc* depended on the dominance relationship between *n* and *c* alleles indicated by parameter *k*: *b*_m_=1−*k* for *nc* males, and *b*_f_=1−*k*(1−*b*) for *nc* females.

We used parameter *α* (0≤*α*≤1) to simulate a mixed sexual/asexual strategy for heterozygotic genotypes, *as*, in the *S* locus. Parameter *α* represents the proportion of individuals of these genotypes that, irrespective of being generated by a sexual or an asexual strategy, reproduce using a sexual strategy, the rest 1−*α* being the proportion of asexual ones. Therefore, we assumed that 1−*α* proportion of individuals of genotypes (*as* *nn*), (*as* *nc*) and (*as* *cc*) used asexual reproductive strategies (*α*=1, 0 or 0.5, when allele *a* is recessive, dominant or codominant, respectively, with respect to allele *s*).

For sexual individuals, differences in male mating success between genotypes were addressed by two positive functions that depended on allelic frequencies at the *M* locus, *η* and *π* (*η*≥0 and 0≤*π*≤1, see below). Functions 1+*η* and 1−*π* represented the mean number of mates for homozigotic *cc* (polygynous) and *nn* (monogamous) males, respectively. Therefore, the mean numbers of mates were represented as deviations from unity. The mean mating success of heterozigotic *nc* males was *θ*=1−*π*(1−*k*)+*ηk*, and thus 1+*η*≤*θ*≤1−*π* depending on the type of dominance relationship between *n* and *c* alleles determined by parameter *k*.

The ability in mating competition was assumed to be higher for polygynous *cc* males compared with monogamous *nn* males, and hence the presence of polygynous males reduced the mating success of monogamous ones below unity. Therefore, depending on phenotype frequencies of male types in the population, 1≤1+*η*≤*m* and 0≤1−*π*≤1 for polygynous and monogamous males respectively, where *m* (*m*≥1) represents the maximum potential number of mates for *cc* males (only achieved when polygynous males are scarce in a population composed mainly of monogamous males). Thus, mean mating success for polygynous males ranged from *m* (when polygynous males are rare) to 1 (when monogamous males are rare), and for monogamous males it ranged from 1 (when polygynous males are rare) to 0 (when monogamous males are rare).

We computed mating functions 1+*η*, *θ* and 1−*π*, assuming that all females in the population mated, as follows.

Let *P*_i_ (when *i* varies from 1 to 9) be the frequencies of genotypes (*G*_1_=*aa*, *nn*), (*G*_2_=*aa*, *nc*), (*G*_3_=*aa*, *cc*), (*G*_4_=*as*, *nn*), (*G*_5_=*as*, *nc*), (*G*_6_=*as*, *cc*), (*G*_7_=*ss*, *nn*), (*G*_8_=*ss*, *nc*) and (*G*_9_=*ss*, *cc*), respectively. *P*_1_, *P*_2_ and *P*_3_ and 1−*α* proportion of *P*_4_, *P*_5_ and *P*_6_ represent the frequencies of asexual individuals in the population. Thus, the proportion of sexual individuals in the population is

2.4 $$\tau =\alpha (P_{4}+P_{5}+P_{6})+(P_{7}+P_{8}+P_{9}),$$

half of them being females. Thus, the accumulated proportion of mates for all males must equal the total proportion of females in the population, *τ*/2:

2.5 $$\frac{1}{2}(\alpha P_{4}+P_{7})(1-\pi )+\frac{1}{2}(\alpha P_{5}+P_{8})\theta +\frac{1}{2}(\alpha P_{6}+P_{9})(1+\eta )=\frac{1}{2}\tau .$$

Then

2.6 $$\eta [\alpha P_{6}+P_{9}+k(\alpha P_{5}+P_{8})]=\pi [\alpha P_{4}+P_{7}+(1-k)(\alpha P_{5}+P_{8})].$$

In the limit, when the maximum polygynous potential is m→∞, only polygynous males can reproduce and monogamous males have no access to females. Then

2.7 $$\underset{m\to \infty }{\lim\eta }=\frac{\alpha P_{4}+P_{7}+(1-k)(\alpha P_{5}+P_{8})}{\alpha P_{6}+P_{9}+k(\alpha P_{5}+P_{8})}$$

and

2.8 $$\underset{m\to \infty }{\lim\pi }=1.$$

A solution that satisfies equations (2.7) and (2.8) is

2.9 $$\eta =\frac{\left(m-1)[\alpha P_{4}+P_{7}+(1-k)(\alpha P_{5}+P_{8})\right]}{1+(m-1)[\alpha P_{6}+P_{9}+k(\alpha P_{5}+P_{8})]}$$

and

2.10 $$\pi =\frac{\left(m-1)[\alpha P_{6}+P_{9}+k(\alpha P_{5}+P_{8})\right]}{1+(m-1)[\alpha P_{6}+P_{9}+k(\alpha P_{5}+P_{8})]}.$$

Therefore, the mean number of mates per male in relation to his genotype in the *M* locus and the dominance relationship between *n* and *c* alleles is

2.11 $$\begin{array}{rl} 1+\eta & =\frac{1+k\tau (m-1)}{1+(m-1)[\alpha P_{6}+P_{9}+k(\alpha P_{5}+P_{8})]}, \end{array}$$

2.12 $$\begin{array}{rl} \theta & =\frac{1+k\tau (m-1)}{1+(m-1)[\alpha P_{6}+P_{9}+k(\alpha P_{5}+P_{8})]} \end{array}$$

2.13 $$\begin{array}{rl} \text{and}\;1-\pi & =\frac{1}{1+(m-1)[\alpha P_{6}+P_{9}+k(\alpha P_{5}+P_{8})]}, \end{array}$$

where 1+*η*, *θ* and 1−*π* being decreasing functions with the frequency of the allele *c* in the population.

The composition of the filial generation in the *t*+1 generation arises through the reproductive effects of all sexual and asexual individuals in the *t* generation. Asexual individuals are the homozygotic ones *aa* at the *R* locus and a proportion 1−*α* of heterozygotic individuals *as*. The asexual contribution to filial genotypes, *L*_*Ai*_ (*i*=1–6), is obtained by replicating the asexual genotype with fitness equal to unity (table 2):

2.14 $$L_{Ai}=P_{i},\;\text{for homozygotes }\text{aa}$$

**Table 2.** Genetic contribution of asexual individuals to the next generation. (Only a proportion 1−*α* of heterozigotic individuals in the *R* locus (*as –*) choose a parthenogenetic strategy *S*_1_. The genotype of the filial generation is an exact copy of the parental generation. The asexual contribution to filial genotypes are *L*_*Ai*_=*P*_i_ when *i*=1, 2 or 3 and *L*_*Ai*_=(1−*α*)*P*_i_ when *i*=4, 5 or 6.)

			filial segregation					
parental genotype	proportions	fitness	return *aa nn*	*aa nc*	*aa cc*	*as nn*	*as nc*	*as cc*
*aa nn*	*P*_1_	1	1	0	0	0	0	0
*aa nc*	*P*_2_	1	0	1	0	0	0	0
*aa cc*	*P*_3_	1	0	0	1	0	0	0
*as nn*	(1−*α*)*P*_4_	1	0	0	0	1	0	0
*as nc*	(1−*α*)*P*_5_	1	0	0	0	0	1	0
*as cc*	(1−*α*)*P*_6_	1	0	0	0	0	0	1
asexual contribution			*L*_*A*1_	*L*_*A*2_	*L*_*A*3_	*L*_*A*4_	*L*_*A*5_	*L*_*A*6_

and

2.15 $$L_{Ai}=(1-\alpha )P_{i},\;\text{for heterozygotes }\text{as}.$$

The proportion of descendants generated by a strategy *S*_1_ is 1−*τ*. However, this value must be corrected by changes in proportions of genotypes owing to the action of sexual selection (competition for mates) operating in sexual strategies (table 3; see below).

**Table 3.** Genetic contribution of sexual organisms to the filial generation, *τ* (equation 3.4 in the text) is the relative proportion of sexual individuals in the population. (*L*_*Si*_ are the sum of all sexual contributions to each filial genotype (see text for details).)

				filial segregation								
parental genotype male×female	male mating	proportions	fitness return	*G*_1_	*G*_2_	*G*_3_	*G*_4_	*G*_5_	*G*_6_	*G*_7_	*G*_8_	*G*_9_
*G*_4_×*G*_4_	1−*π*	$\alpha^{2}P_{4}^{2}$/2*τ*	2*R*	1/4	0	0	1/2	0	0	1/4	0	0
*G*_4_×*G*_5_	1−*π*	*α*^2^*P*_4_*P*_5_/2*τ*	*R*[2−*k*(1−*b*)]	1/8	1/8	0	1/4	1/4	0	1/8	1/8	0
*G*_4_×*G*_6_	1−*π*	*α*^2^*P*_4_*P*_6_/2*τ*	*R*(1+*b*)	0	1/4	0	0	1/2	0	0	1/4	0
*G*_4_×*G*_7_	1−*π*	*αP*_4_*P*_7_/2*τ*	2*R*	0	0	0	1/2	0	0	1/2	0	0
*G*_4_×*G*_8_	1−*π*	*αP*_4_*P*_8_/2*τ*	*R*[2−*k*(1−*b*)]	0	0	0	1/4	1/4	0	1/4	1/4	0
*G*_4_×*G*_9_	1−*π*	*αP*_4_*P*_9_/2*τ*	*R*(1+*b*)	0	0	0	0	1/2	0	0	1/2	0
*G*_5_×*G*_4_	*θ*	*α*^2^*P*_4_*P*_5_/2*τ*	*R*(2−*k*)	1/8	1/8	0	1/4	1/4	0	1/8	1/8	0
*G*_5_×*G*_5_	*θ*	$\alpha^{2}P_{5}^{2}$/2*τ*	*R*[2−*k*−*k*(1−*k*)(1−*b*)]	1/16	1/8	1/16	1/8	1/4	1/8	1/16	1/8	1/16
*G*_5_×*G*_6_	*θ*	*α*^2^*P*_5_*P*_6_/2*τ*	*R*[1+*b*(1−*k*)]	0	1/8	1/8	0	1/4	1/4	0	1/8	1/8
*G*_5_×*G*_7_	*θ*	*αP*_5_*P*_7_/2*τ*	*R*(2−*k*)	0	0	0	1/4	1/4	0	1/4	1/4	0
*G*_5_×*G*_8_	*θ*	*αP*_5_*P*_8_/2*τ*	*R*[2−*k*−*k*(1−*k*)(1−*b*)]	0	0	0	1/8	1/4	1/8	1/8	1/4	1/8
*G*_5_×*G*_9_	*θ*	*αP*_5_*P*_9_/2*τ*	*R*[1+*b*(1−*k*)]	0	0	0	0	1/4	1/4	0	1/4	1/4
*G*_6_×*G*_4_	1+*η*	*α*^2^*P*_4_*P*_6_/2*τ*	*R*	0	1/4	0	0	1/2	0	0	1/4	0
*G*_6_×*G*_5_	1+*η*	*α*^2^*P*_5_*P*_6_/2*τ*	*R*	0	1/8	1/8	0	1/4	1/4	0	1/8	1/8
*G*_6_×*G*_6_	1+*η*	$\alpha^{2}P_{6}^{2}$/2*τ*	*R*	0	0	1/4	0	0	1/2	0	0	1/4
*G*_6_×*G*_7_	1+*η*	*αP*_6_*P*_7_/2*τ*	*R*	0	0	0	0	1/2	0	0	1/2	0
*G*_6_×*G*_8_	1+*η*	*αP*_6_*P*_8_/2*τ*	*R*	0	0	0	0	1/4	1/4	0	1/4	1/4
*G*_6_×*G*_9_	1+*η*	*αP*_6_*P*_9_/2*τ*	*R*	0	0	0	0	0	1/2	0	0	1/2
*G*_7_×*G*_4_	1−*π*	*αP*_4_*P*_7_/2*τ*	2*R*	0	0	0	1/2	0	0	1/2	0	0
*G*_7_×*G*_5_	1−*π*	*αP*_5_*P*_7_/2*τ*	*R*[2−*k*(1−*b*)]	0	0	0	1/4	1/4	0	1/4	1/4	0
*G*_7_×*G*_6_	1−*π*	*αP*_6_*P*_7_/2*τ*	*R*(1+*b*)	0	0	0	0	1/2	0	0	1/2	0
*G*_7_×*G*_7_	1−*π*	$P_{7}^{2}$/2*τ*	2*R*	0	0	0	0	0	0	1	0	0
*G*_7_×*G*_8_	1−*π*	*P*_7_*P*_8_/2*τ*	*R*[2−*k*(1−*b*)]	0	0	0	0	0	0	1/2	1/2	0
*G*_7_×*G*_9_	1−*π*	*P*_7_*P*_9_/2*τ*	*R*(1+*b*)	0	0	0	0	0	0	0	1	0
*G*_8_×*G*_4_	*θ*	*αP*_4_*P*_8_/2*τ*	*R*(2−*k*)	0	0	0	1/4	1/4	0	1/4	1/4	0
*G*_8_×*G*_5_	*θ*	*αP*_5_*P*_8_/2*τ*	*R*[2−*k*−*k*(1−*k*)(1−*b*)]	0	0	0	1/8	1/4	1/8	1/8	1/4	1/8
*G*_8_×*G*_6_	*θ*	*αP*_6_*P*_8_/2*τ*	*R*[1+*b*(1−*k*)]	0	0	0	0	1/4	1/4	0	1/4	1/4
*G*_8_×*G*_7_	*θ*	*P*_7_*P*_8_/2*τ*	*R*(2−*k*)	0	0	0	0	0	0	1/2	1/2	0
*G*_8_×*G*_8_	*θ*	$P_{8}^{2}$/2*τ*	*R*[2−*k*−*k*(1−*k*)(1−*b*)]	0	0	0	0	0	0	1/4	1/2	1/4
*G*_8_×*G*_9_	*θ*	*P*_8_*P*_9_/2*τ*	*R*[1+*b*(1−*k*)]	0	0	0	0	0	0	0	1/2	1/2
*G*_9_×*G*_4_	1+*η*	*αP*_4_*P*_9_/2*τ*	*R*	0	0	0	0	1/2	0	0	1/2	0
*G*_9_×*G*_5_	1+*η*	*αP*_5_*P*_9_/2*τ*	*R*	0	0	0	0	1/4	1/4	0	1/4	1/4
*G*_9_×*G*_6_	1+*η*	*αP*_6_*P*_9_/2*τ*	*R*	0	0	0	0	0	1/2	0	0	1/2
*G*_9_×*G*_7_	1+*η*	*P*_7_*P*_9_/2*τ*	*R*	0	0	0	0	0	0	0	1	0
*G*_9_×*G*_8_	1+*η*	*P*_8_*P*_9_/2*τ*	*R*	0	0	0	0	0	0	0	1/2	1/2
*G*_9_×*G*_9_	1+*η*	$P_{9}^{2}$/2*τ*	*R*	0	0	0	0	0	0	0	0	1
sexual contribution				*L*_*S*1_	*L*_*S*2_	*L*_*S*3_	*L*_*S*4_	*L*_*S*5_	*L*_*S*6_	*L*_*S*7_	*L*_*S*8_	*L*_*S*9_

The contribution of sexual individuals to the filial generation includes a chain of effects: (i) proportion of male–female pairs per genotype and mean number of mates by males of this genotype, (ii) differences in fitness of male–female pairs, and finally, (iii) filial segregation of these pairs. The sexual contribution to the filial genotype *i*, *L*_*Si*_ (*i*=1–9), is the sum of the effects of pairs that segregate into this genotype. However, these values need to be corrected after the sexual selection and asexual reproduction processes have operated (table 3).

For sexual strategies, 36 male × female crossing genotypes are possible (combinations of *as nn*=*G*_4_, *as nc*=*G*_5_, *as cc*=*G*_6_, *ss nn*=*G*_7_, *ss nc*=*G*_8_ and *ss cc*=*G*_9_ genotypes; table 3). We do not consider departures from random mating (i.e. panmixia) other than differences in the degree of polygyny between males in relation to their genotypes. Two offspring are produced by each sexual pair, with recombined (Mendelian) genetic material and even sex ratio. The proportion of each of the 36 combinations of male × female pairs can be generated by the terms of a squared polynomial:

2.16 $$\frac{1}{2\tau }{\left[\alpha (P_{4}+P_{5}+P_{6})+P_{7}+P_{8}+P_{9}\right]}^{2}=\frac{\tau }{2}.$$

The relative contribution for each male × female pair to the next generation can be determined by the proportion of this combination in the population (term in the squared polynomial, equation (2.16)), its fitness (equation (2.2)), the mean number of mates of the male in relation to his genotype (equations (2.11), (2.12) and (2.13)) and the Mendelian segregation of genotypes (table 3). For example, the proportion of pairs with *as nc* male (*θ* being its mean number of mates, equation (2.12)) and *as cc* female (i.e. *G*_5_×*G*_6_ mating in table 3) is

2.17 $$\frac{1}{2\tau }\alpha^{2}P_{5}P_{6}.$$

Thus, the fitness of their descendants (equation (2.2)) is

2.18 R[1+b(1−k)].

Finally, after Mendelian segregation of genotypes, the relative contribution of *G*_5_×*G*_6_ pairs for each of their six types of offspring genotypes (*G*_2_,*G*_3_, *G*_5_, *G*_6_, *G*_8_ and *G*_9_, see table 3) is 1/4 or 1/8 of the expression

2.19 $$\frac{\alpha^{2}P_{5}P_{6}R[1+b(1-k)]\theta }{2\tau }.$$

The frequencies for the nine possible genotypes in the *t*+1 generation can be obtained after a two-step process. First, we add all relative contributions coming from sexual and asexual strategies, *L*_*Ai*_(*t*) and *L*_*Si*_(*t*). Let us now consider *L*_i_(*t*)=*L*_*Ai*_(*t*)+*L*_*Si*_(*t*), *i*=1,2,…9, the sum of all partial contributions (tables 2 and 3) to filial genotypes. However, owing to the action of the sexual selection pressures, the sum of all contributions to the nine filial genotypes does not equal 1. Second, we correct *L*_i_(*t*) proportions to obtain the new genotype frequencies:

2.20 $$P_{i}(t+1)=\frac{L_{i}(t)}{\sum_{j=1}^{9}Lj(t)},$$

where $\sum_{i=1}^{9}P_{i}(t+1)=1.$ The new allelic frequencies are

2.21 $$\begin{array}{rl} a(t+1) & =\sum_{i=1}^{3}P_{i}(t+1)+\frac{1}{2}\sum_{i=4}^{6}P_{i}(t+1), \end{array}$$

2.22 $$\begin{array}{rl} s(t+1) & =1-a(t+1), \end{array}$$

2.23 $$\begin{array}{rl} n(t+1) & =\sum_{i=3,6,9}P_{i}(t+1)+\frac{1}{2}\sum_{j=2,5,8}P_{j}(t+1) \end{array}$$

2.24 $$\begin{array}{rl} \text{and}\;c(t+1) & =1-n(t+1). \end{array}$$

Equations (2.21)–(2.24) allow us to explore the evolutionary stability of both single and mixed strategies in the population.

## 3. Results

We derived analytical solutions when feasible and carried out computer simulations to study the fate of allele mutants or migrants when introduced in a population where other alleles predominated. Allele frequencies increased/decreased according to fitness functions, with parameter values corresponding to the behaviour of the interacting strategists. We first explored the interaction for pairs of strategies; that is, when one of the alleles was not present in the population (i.e. strategies *S*_1_ versus *S*_2_; *S*_1_ versus *S*_3_; and *S*_2_ versus *S*_3_). Thereafter, we investigated the interaction between all the strategies when the four alleles at both *R* and *M* loci were present. Simulations were run for the relevant range of *R* (1–2) and *m* (≥2) parameters. Variations in initial conditions for *α*,*k* and *b* as well as in initial allele frequencies were explored but they are not fully presented here for simplicity. Interested readers can explore themselves all desired variations in the provided R script (see Data accessibility section).

### 3.1 Asex versus monogamous, non-costly sex

Here we consider the competition between non-costly sex (strategy *S*_2_) and asexual reproduction (strategy *S*_1_). For this particular case, allele frequency of the costly sex in the *M* locus is *c*=0 and fitness return (equation (2.2)) becomes *W*_s_=2*R*. The frequency of the allele *a* between two successive generations, *t* and *t*+1, has an analytical solution:

3.1 $$a(t+1)=\frac{a(t)+1\text{/}2\alpha (R-1)P_{4}(t)}{1+(R-1)\tau (t)},$$

where *τ*(*t*)=*αP*_4_+*P*_7_ (see equation (2.4)) is the proportion of sexual individuals in the parental generation *t*. Equation (3.1) has three equilibrium solutions: (i) pure-asexual strategy *S*_1_ (i.e. allele frequency *a*=1); (ii) pure non-costly (monogamous) sexual strategy *S*_2_ (i.e. allele frequency *s*=1); and (iii) equilibrium between non-costly sex and asexual strategy (*S*_1_/*S*_2_), irrespective of all possible values of allelic frequencies *a* and *s*, when *R*=1. Thus, the only possible evolutionarily stable strategies (ESSs) are asexuality (*S*_1_) when *R*<1 and monogamous sex (*S*_2_) when *R*>1, or an equilibrium between asexuality and monogamous sex (*S*_1_/*S*_2_), for all possible values of alleles *a* and *s*, when *R*=1.

**Figure 1.**
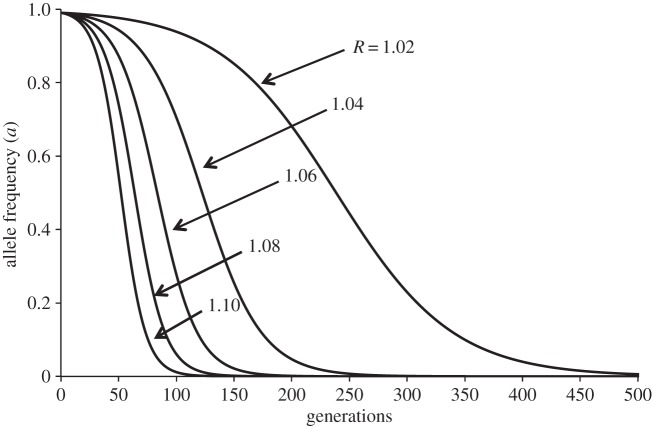
Changes in the frequency of allele *a* throughout generations when non-costly sexual mutants (i.e. with alleles *s* and *n*) are introduced into a pure population of asexual individuals, for different values of the benefit of recombination *R*. The higher the *R* the more rapid is the change from strategy *S*_1_ to evolutionarily stable strategy *S*_2_. The initial frequency values for the simulations were *a*=0.99 and *s*=0.01 (with *n*=1 and *c*=0).

The dynamics of the invasion of a pure-asexual population *S*_1_ by monogamous *s* mutants is dependent on the value of parameter *R*; the higher *R* the more speedy is the change from *S*_1_ to the ESS *S*_2_ (figure 1).

### 3.2 Asex versus polygynous, costly sex

Let us now consider the interaction between costly sex (*S*_3_) and asexual reproduction (*S*_1_). For this case, allele frequency of the costly sex in the *M* locus is *c*=1 and fitness return (equation (2.2)) becomes *W*_s_=*R*. The problem is similar to the previous *S*_1_ versus *S*_2_ and also has an analytical solution:

3.2 $$a(t+1)=\frac{a(t)+1\text{/}4\alpha (R-2)P_{6}(t)}{1+1\text{/}2(R-2)\tau (t)},$$

where *τ*(*t*)=*αP*_6_+*P*_9_ (see equation (2.4)) is the proportion of sexual individuals in the parental generation *t*; *P*_6_ and *P*_9_ being the proportions of genotypes *as cc* and *ss cc*, respectively, in the same generation *t*. Equation (3.2) has the following possible solutions: (i) pure-asexual strategy *S*_1_ (i.e. allele frequency *a*=1); (ii) pure costly sex strategy *S*_3_ (i.e. allele frequency *s*=1); and (iii) equilibrium between costly sex and asexual strategy (S_1_/*S*_3_), irrespective of all possible values of allele frequencies *a* and *s*, when *R*=2. Parameters *m* and *b* have no effect here as all sexual individuals are equally competitive, hence mean number of mates (equation (2.11)) becomes 1+*η*=1.

ESSs are asexuality (*S*_1_) when *R*<2, polygynous sex (*S*_3_) when *R*>2, and the equilibrium between asexuality and polygynous sex (*S*_1_/*S*_3_), for all possible values of alleles *a* and *s*, when *R*=2. This result is in agreement with the common view that costly sex needs twofold benefits of recombination to spread in a population of asexuals. As for the previous *S*_1_ versus *S*_2_ problem, the dynamics of the invasion of a pure-asexual population *S*_1_ by polygynous *c* mutants is dependent on the value of parameter *R*; the higher the value of *R* above 2 the more speedy is the change from *S*_1_ to the ESS *S*_3_.

### 3.3 Monogamous versus polygynous sex

The interaction between costly (*S*_3_) and non-costly (*S*_2_) sex is represented by *c* and *n* alleles, when asexuals are not present in the population (i.e. *s*=1 in *S* locus and *τ*=1). The mean number of mates of homozygote polygynous *cc* males and those of heterozygote polygynous *cn* males compared to monogamous homozygote *nn* males are *m* and 1+*k*(*m*−1), respectively (from equation (2.11–2.13).

The evolutionary game between these two strategies is analysed elsewhere (Polo & Carranza [60]). Analytical solutions are possible at both extremes of the dominance relationship between alleles *n* and *c*, and only numeric solutions for intermediate values of dominance between alleles *a* and *c*, (i.e. when 0<*k*<1). The game drive model shows that the mating potential for polygynous males, *m*, is the most important factor explaining the dynamics of the competition between *n* and *c* alleles, and that *k*, and the proportion of male parental care accepted by females, *b*, have secondary, although important effects (see below the interaction among the three strategies).

In summary, the model for the paired interaction between both sexual strategies shows: (i) when allele *n* is dominant or recessive (*k*=0 or 1, respectively) non-costly sex (*S*_2_) is the only ESS if *m*<1+*b*, costly sex (*S*_3_) is the ESS if *m*≥3−*b*, and (*S*_2_) or (*S*_3_) prevail depending on the initial frequencies of *n* and *c* if 1+*b*≤*m*<3−*b*; and (ii) when alleles *c* and *n* have intermediate values of dominance relationship a third region appears composed of an equilibrium between non-costly and costly strategies (*S*_2_/*S*_3_), for all possible values of alleles *n* and *c*, when there are intermediate values of *m* and high values of *b* (details in Polo & Carranza [60]).

### 3.4 Asex versus monogamous versus polygynous sex

When the four alleles at both loci were present, our results confirmed that asexuality, *S*_1_, was the only ESS when the relative benefit of recombination was *R*<1, and that for high values of the relative benefit of recombination (*R*>2), parthenogenetic individuals were replaced by either costly or non-costly sexual ones, the interaction between both sexual strategies depending again on parameters *m*, *k*, *b* and initial allele frequencies.

Indeed, the most interesting results are those for intermediate values of the relative benefit of recombination, 1<*R*<2. For this situation we found that the dynamics of the three strategies (*S*_1_, *S*_2_, *S*_3_) that resulted from the combined effects of alleles *a*, *s*, *n* and *c*, represented a rock–paper–scissors' type model based on the following, intransitive fitness inequalities between strategies: *W*(*S*_2_)>*W*(*S*_1_) (as *R*>1; see §3.1 above); *W*(*S*_1_)>*W*(*S*_3_) (as *R*<2; see §3.2 above) and *W*(*S*_3_)>*W*(*S*_2_) when *m* was above a minimum value, also depending on *b* and *k* parameters (see §3.3 above).

**Figure 2.**
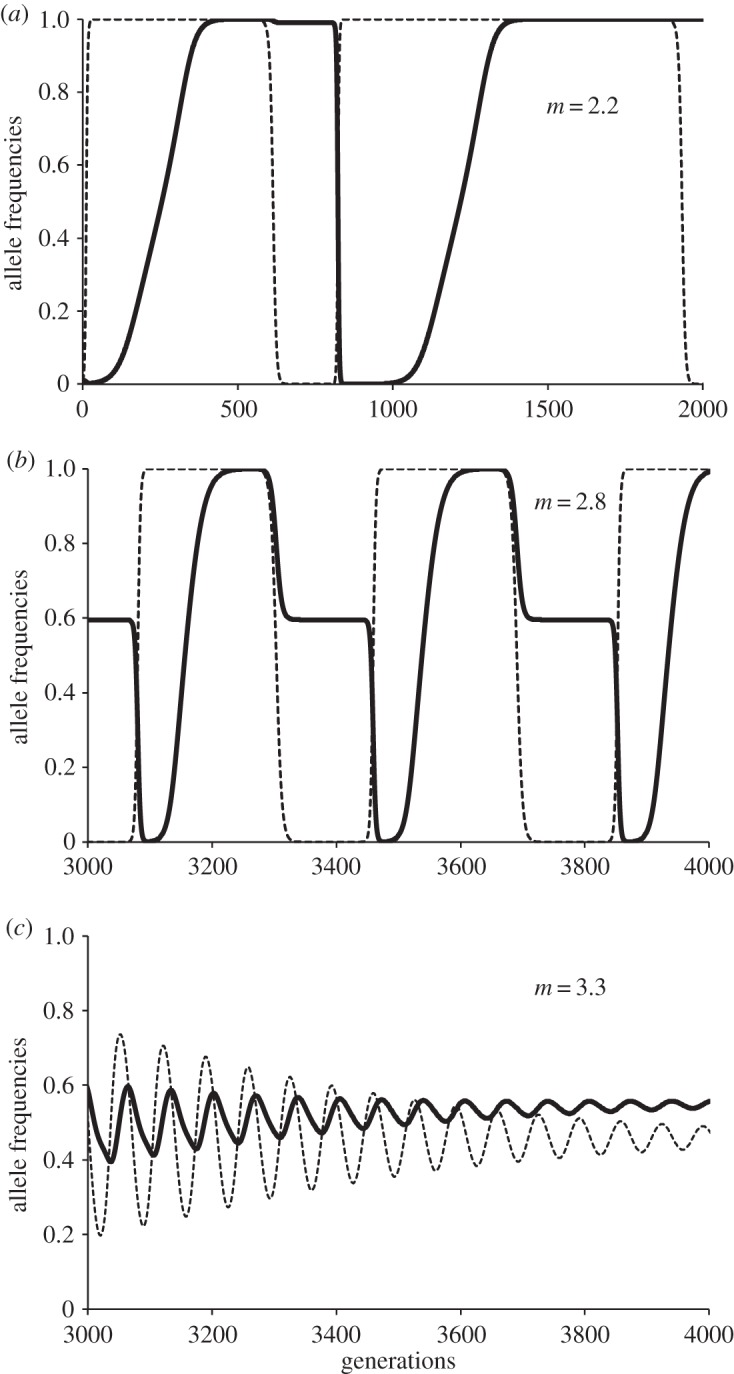
Examples of dynamics for alleles *c* (thick line) and *s* (thin line), with parameter values *α*=*b*=*k*=0.5, very low initial frequencies of *s* and *c* (0.01), *R*=1.6 and three values for *m*=2.2 (*a*), 2.8 (*b*) and 3.3 (*c*).

**Figure 3.**
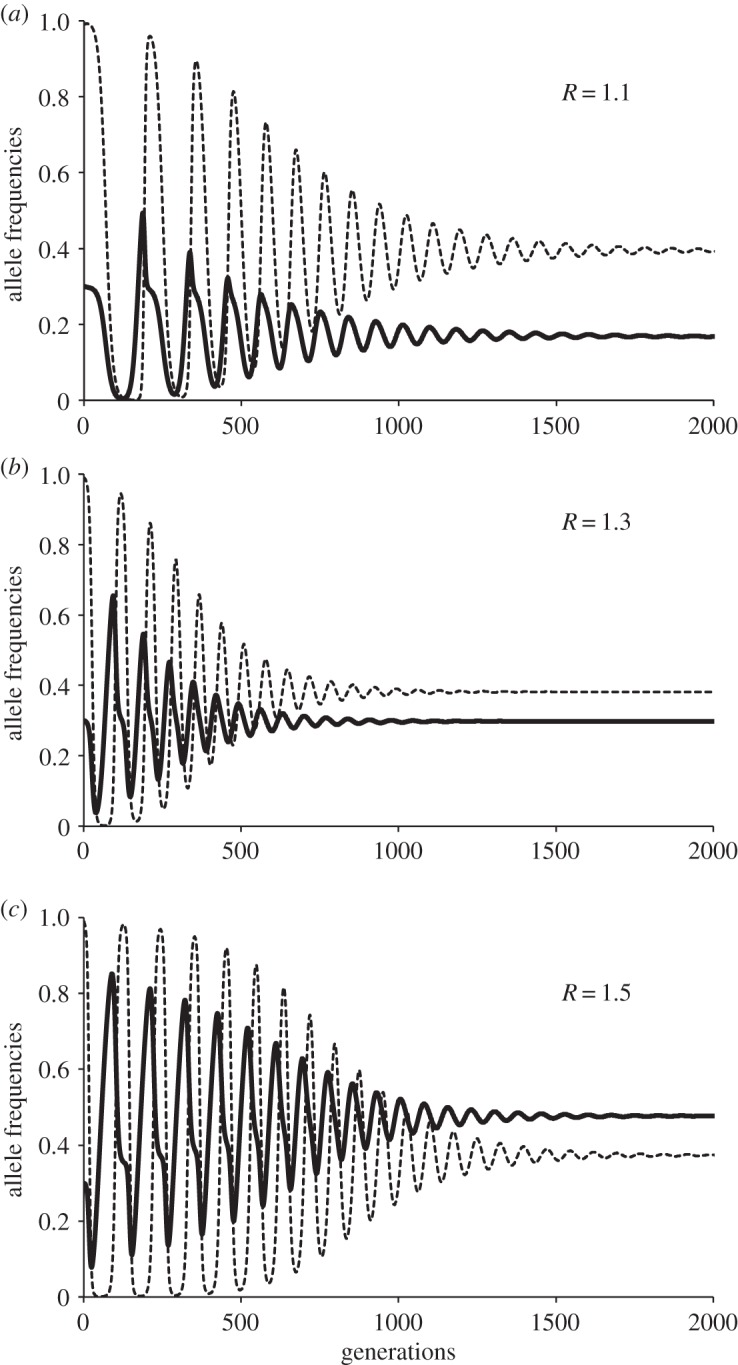
Effects of *R* variation: cyaboveclic dynamics of asexuality (allele *a*: dotted lines) and costly sex (allele *c*: thick lines) for a population with all four alleles at the two loci. The initial frequency values for the simulations were *a*=0.99 and *c*=0.3. Simulations were run for *α*=*k*=*b*=0.5 and *m*=2.7 and three values for the benefits of recombination: (*a*) *R*=1.21, (*b*) *R*=1.3 and (*c*) *R*=1.5. The main effect of *R* increment was the increase of the equilibrium frequency for costly sex allele (*c*=0.168, 0.298 and 0.477, respectively), but not the decrease in the proportion of asexuals in the population.

**Figure 4.**
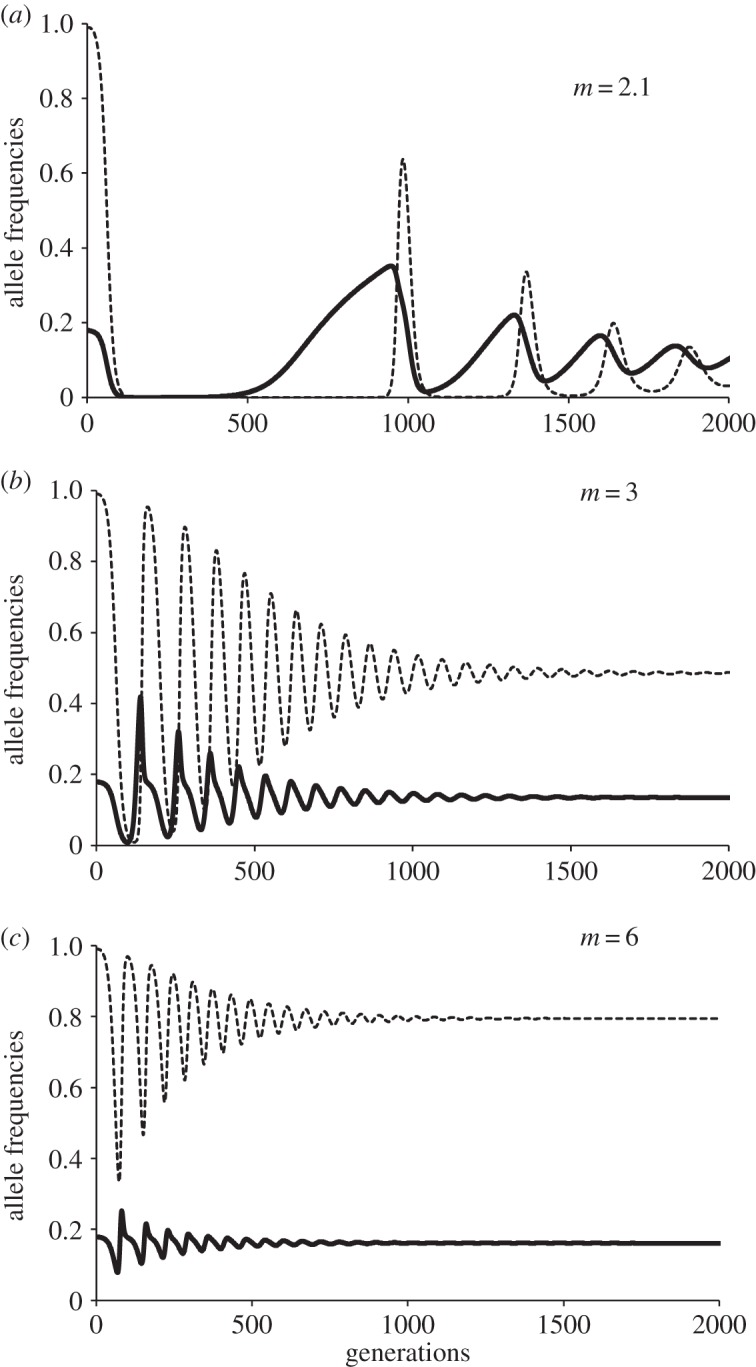
Effects of *m* variation: cyclic dynamics of asexuality (allele *a*: dotted lines) and costly sex (allele *c*: thick lines) for a population with all four alleles at the two loci. The initial frequency values for the simulations were *a*=0.99 and *c*=0.18. Simulation runs for *α*=*k*=*b*=0.5 and *R*=1.1 and three values of the polygyny potential: (*a*) *m*=2.1, (*b*) *m*=3 and (*c*) *m*=6. The main effect of *m* increment was the increase of the frequency of asexuals (*a*=0.031, 0.487 and 0.794, respectively), but only a very small increase in the proportion of costly sex in the population.

To exemplify these interactions, we may observe the dynamics of a population where the parthenogenetic *S*_1_ strategy was dominant but alleles *s*, *n* and *c* were also present (figure 2). The frequency of the allele *a* in locus *S* tended to decrease as non-costly sex *S*_2_ (alleles *s* and *n* in loci *S* and *M*, respectively) increased, because of the inequality *W*(*S*_2_)>*W*(*S*_1_), and then costly sex *S*_3_ (hence alleles *s* and *c* in loci *S* and *M*, respectively) was favoured if *m* is big enough (e.g. *m*=2.2 to 3.3 in figure 2), because of the inequality *W*(*S*_3_)>*W*(*S*_2_). But then, the rise of allele *c*, in turn, favoured asexuality *S*_1,_ because of the inequality *W*(*S*_1_)>*W*(*S*_3_), and then *S*_2_ again. The relative benefit of recombination, *R*, and the polygyny potential, *m*, had important effects on the equilibrium state of strategies *S*_1_/*S*_2_/*S*_3_, allowing a mixed dynamic with the three strategies. In the example of figure 2 with *R*=1.6, the increase of polygyny potential *m* reduced the duration and the amplitude of cycles and increased the predominance of the costly sex allele *c*. Rather surprisingly, the main effect of rising *R* was the increase in the frequency of costly sex allele *c* (figure 3), rather than the decrease in the proportion of asexual individuals in the population. For low values of *R* (e.g. *R*=1.1; figure 4), the main effect of increasing *m* was the increase of asexual individuals along with only small increase in the frequency of the costly sex allele in the population. This result arises under these conditions because higher *m* makes alleles from polygynous males (*c*) spread very quickly at the expense of low-cost alleles *n*. Then, asexuality is not so effectively displaced because: (i) sexual reproduction predominates in its costly form, and (ii) the low *R* value provides very little advantage to sexuals.

**Figure 5.**
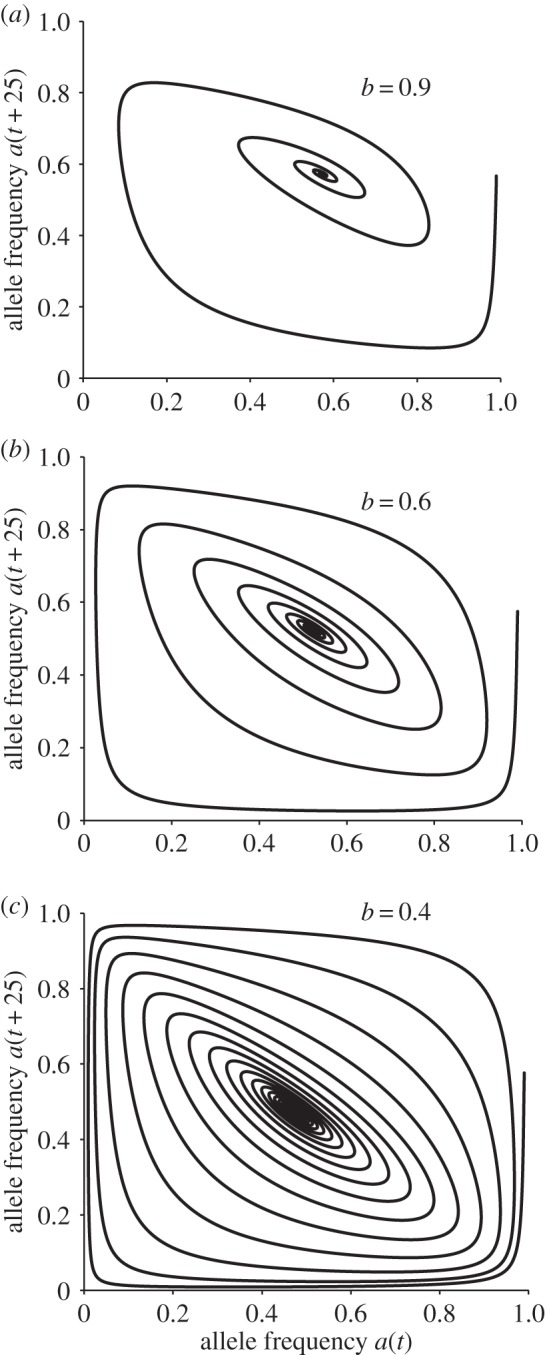
Cyclic dynamics of asexuals, *a*, towards the equilibrium in a mixed strategy *S*_1_/*S*_2_/*S*_3_. We displayed *a*(*t*+25) versus *a*(*t*) to show the transition dynamics towards the attractor point. Simulation runs for *α*=*k*=0.5, *m*=3.1 and *R*=1.3 and three values of the *b* parameter: (*a*) *b*=0.9, (*b*) *b*=0.6 and (*c*) *b*=0.4. The main effect of the decrease in *b* is a high increase of the transitory phase, but a short decrease in the proportion of asexuals.

Other parameters affected more the duration of the transitory phases than the equilibrium balance between strategies. Thus, a decrease in *b* highly increased the transitory phase, and also had a second-order effect of decreasing the final proportion of asexuals at the attractor point (figure 5). On the other hand, deviations from perfect codominace between alleles (i.e. *k*>0.5 and *k*<0.5) increased the cyclic processes, and also had second-order effects on allele frequencies at equilibrium: increase of *k* decreased costly sex with respect to non-costly sex and slightly increased the frequency of asexuals. Finally, the proportion *α* of sexual heterozygote *as* individuals had a negligible effect either in allele frequencies or in the duration of transitory processes.

**Figure 6.**
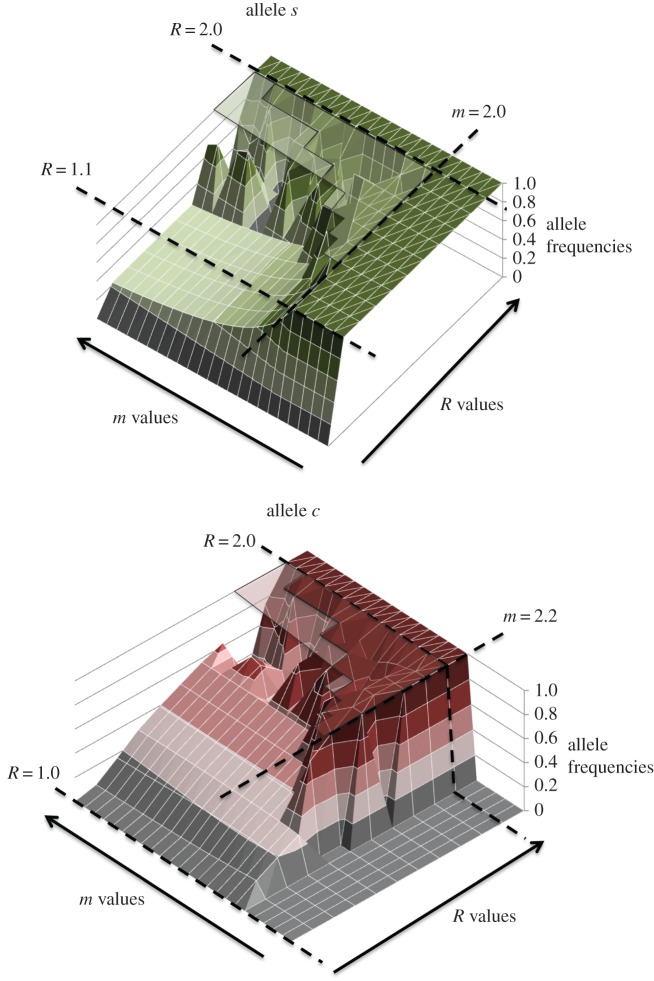
Parameter space of frequencies of alleles *s* and *c* at generation 10 000 for the example in table 4. Figure shows allele frequencies at *t*=10 000. Cells within the parameter space represent cases that reached stable equilibria or instances in continuous oscillations (graph area indicated by ‘*’), some of which (**) reached values very close to fixation or extinction (1 or 0) with only traces of the competing alleles that can resume the cycles thanks to the infinite size of the modelled population (see table 4 for details of cell values, and figure 2 for the dynamics of alleles in three example cases within the parameter space). Variations in conditions can be explored with the provided R script ([61] and the electronic supplementary material).

Parameter space (as the example depicted in table 4 and figure 6 for *α*=*b*=*k*=0.5 and initial frequencies *s*=*c*=0.01) showed that:

— asexuality, *S*_1_, is not an ESS in presence of alternative modes of sexual reproduction when there is some, even very small benefit of recombination (*R*>1);
— non-costly sex, *S*_2_, was the only ESS when 1<*R*<2 and *m*≤2;
— the costly sex allele *c* was never completely eliminated for 1<*R*<2 except when *n*allele fixates at *m*≤2;
— as *R* approaches 2 (approx. above 1.4, table 4) and for *m* values only moderately higher than 2 (approx. below 2.2, table 4) the rock–paper–scissors dynamics showed transitory cycles between regions of dominant asexuality *S*_1_ (*a* approx. 1) followed by a short or non-existent region of dominant non-costly sex *S*_2_ (*s* and *n* approx. 1) and finally by a region of dominant costly sex *S*_3_ (*s* and *c* approx. 1) and then a new *S*_1_ region. In many cases, the dominant region *S*_2_ was not present and the dynamics became mainly a transition between asexuality and costly sex strategies with the amplitude of transitory cycles increasing so that the frequencies of either *a* or *c* alleles may be very close to 1. This means that they would probably fixate in any finite population leading to either pure-asexual or costly sexual populations for *R*>1 and *m*>2, the odds for sexual ones being higher the closer they are to the *R*=2 and *m*=2 corner in the parameter space; and
— most of the parameter space for 1<*R*<2 showed three coexisting strategies *S*_1_, *S*_2_ and *S*_3_ (either in stable equilibrium *S*_1_/*S*_2_/*S*_3_ or in cycles), the main exception being the fixation of non-costly sex allele *n* against costly sex *c* when polygyny potential was low (*m*≤2), rather than the displacement of sexuals by asexuals (figure 6 and table 4; see also figure 2 for the dynamics of some particular cases within this parameter space).

**Table 4.** Parameter space data (i.e. allele frequencies for alleles *s* and *c*) at generation 10 000 as a function of *R* and *m* values, when *α*=*b*=*k*=0.5 and initial frequencies were *s*=*c*=0.01. (Table shows allele frequencies at *t*=10 000. Cells represent cases that reached stable equilibria or instances in oscillations (*), some of which (**) reached values very close to fixation or extinction (1 or 0) with only traces of the competing alleles that can resume the cycles thanks to the infinite size of the modelled population. Note that transitions between these areas within the parameter space are not discrete, so that for some cells near the borders of the equilibrium area allele frequencies can still experience slight variations after generation 10 000, also depending on variations in initial conditions, although in all explored cases within the area without asterisks they finally reached fixation. Likewise, cases with oscillations can reach steady states after enough number of generations, the sooner the closer to the equilibrium area. See also figure 6 and the provided R script ([61] and the electronic supplementary material).)

	*R* values										
*m* values	≤1	1.1	1.2	1.3	1.4	1.5	1.6	1.7	1.8	1.9	≥2
allele *s*											
5.6	0	0.224	0.227	0.228	0.230	0.232	0.230	0.709*	0.019*	0.654*	1
5.4	0	0.234	0.237	0.239	0.240	0.242	0.240	0.768*	0.002*	0.443*	1
5.2	0	0.245	0.248	0.250	0.252	0.254	0.252	0.018*	0.041*	0.578**	1
5.0	0	0.257	0.261	0.263	0.265	0.266	0.264	0.819*	0.002*	0.933**	1
4.8	0	0.271	0.274	0.276	0.279	0.280	0.278	0.864*	0.360*	0.000**	1
4.6	0	0.286	0.290	0.292	0.294	0.296	0.294	0.019*	0.005*	0.060**	1
4.4	0	0.303	0.307	0.309	0.312	0.313	0.311	0.029*	0.016*	0.035**	1
4.2	0	0.322	0.326	0.328	0.331	0.333	0.331	0.031*	0.993*	0.984**	1
4.0	0	0.343	0.347	0.350	0.353	0.355	0.353	0.893*	0.992**	0.992**	1
3.8	0	0.367	0.372	0.375	0.378	0.380	0.378	0.078*	0.935**	0.000**	1
3.6	0	0.396	0.401	0.404	0.407	0.410	0.407	0.738*	0.000**	0.813**	1
3.4	0	0.429	0.434	0.438	0.441	0.444	0.441	0.942**	0.998**	0.015**	1
3.2	0	0.468	0.474	0.478	0.482	0.484	0.481	0.000**	0.000**	0.000**	1
3.0	0	0.514	0.521	0.526	0.530	0.533	0.992*	0.000**	1.000**	0.000**	1
2.8	0	0.572	0.579	0.584	0.589	0.592	1.000**	0.000**	1.000**	0.009**	1
2.6	0	0.643	0.652	0.657	0.663	1.000**	0.000**	1.000**	1.000**	0.000**	1
2.4	0	0.735	0.745	0.752	0.000**	0.000**	1.000**	1.000**	1.000**	1.000**	1
2.2	0	0.857	0.870	0.879	1.000**	1.000**	1.000**	1.000**	1.000**	0.000**	1
≤2	0	1	1	1	1	1	1	1	1	1	1
allele *c*											
5.6	0	0.040	0.164	0.299	0.395	0.481	0.565	0.625*	0.176*	0.748*	1
5.4	0	0.042	0.168	0.300	0.396	0.482	0.566	0.564*	0.172*	0.606*	1
5.2	0	0.043	0.172	0.302	0.396	0.482	0.566	0.314*	0.184*	0.713**	1
5.0	0	0.045	0.176	0.303	0.396	0.482	0.567	0.664*	0.200*	0.956**	1
4.8	0	0.046	0.180	0.304	0.396	0.482	0.567	0.642*	0.501*	0.386**	1
4.6	0	0.048	0.184	0.305	0.396	0.482	0.567	0.308*	0.245*	0.462**	1
4.4	0	0.050	0.188	0.306	0.396	0.482	0.568	0.413*	0.282*	0.464**	1
4.2	0	0.053	0.192	0.306	0.395	0.482	0.568	0.296*	0.766*	0.993**	1
4.0	0	0.055	0.196	0.306	0.395	0.481	0.568	0.858*	0.414**	0.997**	1
3.8	0	0.058	0.199	0.306	0.394	0.481	0.568	0.371*	0.961**	0.666**	1
3.6	0	0.062	0.202	0.305	0.394	0.481	0.568	0.821*	0.492**	0.951**	1
3.4	0	0.066	0.205	0.304	0.393	0.480	0.568	0.954**	0.998**	0.814**	1
3.2	0	0.070	0.206	0.303	0.392	0.480	0.568	0.486**	0.698**	0.875**	1
3.0	0	0.076	0.207	0.301	0.391	0.479	0.893*	0.626**	0.001**	0.924**	1
2.8	0	0.082	0.207	0.299	0.389	0.478	0.984**	0.782**	0.000**	0.957**	1
2.6	0	0.089	0.204	0.297	0.387	1.000**	0.807**	1.000**	1.000**	0.975**	1
2.4	0	0.097	0.201	0.293	0.749**	0.881**	1.000**	1.000**	1.000**	1.000**	1
2.2	0	0.102	0.195	0.284	0.000**	0.000**	1.000**	0.000**	1.000**	1.000**	1
≤2	0	0	0	0	0	0	0	0	0	0	0

## 4. Discussion

Our model can provide an answer to the old, persistent question of why sex is not displaced by asexuality in the short term: the diversity of sexual breeding systems that can occur during the evolution of a lineage, with variable degrees in the twofold cost-of-sex and in the opportunity for sexual selection, prevent the invasion and fixation of asexuality. In particular, our results show that with very small benefits of recombination (well below twofold), alleles promoting costly sex cannot be displaced by those promoting asexuality if sexual reproduction is represented by two modes that correspond to breeding systems commonly occurring in nature, such as monogamy and polygyny, which can frequently occur as flexible options even within a population (e.g. [62–69]; see below).

Our model does not deal with the nature of the benefits of recombination. Rather, it simply explores a scenario in which recombination may be assumed to provide some benefit in the short term, although not necessarily twofold relative to asexuality. This is important in formulating the problem of sex [1–4]: some benefit of recombination may be necessary at the origin of sexual reproduction [70], but a twofold advantage (either from single or multiple causes: [12]) may not be required for costly sex to be maintained.

The three strategies included in our model have intransitive fitness relationships that follow the conditions of the rock–paper–scissors game [71,72], so that all the alleles involved can be maintained either in equilibrium or cycling depending on parameter conditions. This situation can solve the question of why sex is not displaced by asexuality before long-term effects have time enough to accumulate.

Besides the presence of the three strategies, the remaining conditions in our approach are rather unfavourable for the maintenance of costly sex. For example, populations in nature are not infinite, and finite populations have been proved to favour the persistence of sex [14,15,53,54]. In our model there are no density-dependent effects, and density may favour sex against clones [17]. We assumed global dispersion of individuals throughout the population while local dispersion and geographical structure can make sexuals to resist asexual invasions [73].

Our results for pairs of strategies simply confirm previous findings of models that investigated sex versus non-sex [4,14,21,24,46]. Non-costly sex and asexuality can maintain in equilibrium when there is no net benefit of recombination (*R*=1), irrespective of all possible values of allele frequencies *a* and *n*; but non-costly sex is the only ESS when 2>*R*>1. Likewise, a pure costly sex strategy (*S*_3_) needs twofold benefits of recombination to spread when only asexual individuals are present in the population.

However, when alleles promoting the three basic strategies are allowed to interact, our model does not support the classical assumption that asexuality can replace sexual reproduction when recombination does not compensate for the twofold cost of sex [1–3,11]. Rather, the nature of the problem of the maintenance of sex appears to be cyclical, simply by considering the intransitive interactions between reproductive modes that may vary with respect to the twofold cost instead of the contest between only two extremes as in the original proposal.

With some value of *R* not so high as to compensate the twofold cost, variations in the polygyny potential *m* can largely influence the dynamics of the interaction between alleles promoting different reproductive strategies. Polygyny potential provides advantages to *c* alleles with respect to *n* alleles, causing a cycling dynamic between the twofold cost, non-costly sex and the asexuality tending to a continuous rock–paper–scissors game or to equilibrium between alleles. Therefore, an important result in our model is that such prevalence of asexuality caused by the twofold cost of polygyny does not completely eliminate the sexual strategies whenever any non-costly sexual mode remains as a possible alternative to proliferate in the long term. Although reproductive modes such as fair hermaphroditism, monoecy, nuptial gifts or biparental care are acknowledged to reduce the twofold cost of sex ([2,5], but see [74]), their role in the maintenance of sexual reproduction has been much overlooked. Our model shows that these strategies that do not include the full twofold cost of sex may be crucial for the evolutionary maintenance of polygynous sex because they can buffer the evolutionary interactions between asexual and costly sexuals.

Data for current polygynous systems reflect that successful males can inseminate many females without contributing to parental care (see e.g. [10,75]), but a gradualistic variation occurs in many systems with reducing paternal care while increasing mating effort. For example, a proportion of multiple inseminations by unidirectional sperm transfer are present in most hermaphrodites [70,77], and males of most monogamous species engage in extrapair copulations [78] at the expense of reducing their contribution to parental care [10,79]. For a number of organisms the closest alternative to costly sex is not asexuality but biparental care. Female preference for good genes versus paternal investment represents the flexible balance at the individual level between fecundity (by paternal contribution) and costly sex (with stronger selection on male mating ability) [10,58,59,80]. Displacements along the gradient between low-cost and more-costly sex may have repeatedly occurred during the evolution of any lineage, for example by adjusting the mating system. Temporal and spatial variations in ecological conditions in the distribution range of a species promote the variability of mating systems (e.g. [69,81]), and phylogenies reflect that changes in mating systems should have occurred during the evolution of lineages, affecting the gains and losses of sexual traits [82].

Changes between breeding/mating strategies may not be equally probable. Our model has treated all changes equally, although in nature the maintenance of a reproductive strategy against invasion by alternative strategies may not only depend on parameter conditions but also on the ease of shifting between strategies, which is also likely to be different according to the direction of change. Thus, the step from asexuality to sexual reproduction can still occur in nature [66], and most predominately asexual organisms keep some sexual functioning [34,67,68,83,84]. Displacements between low-cost and costly sex can be achieved in both directions simply by changing the mating system and the relative contribution of the sexes to parental care (e.g. [10,85]). By contrast, mutations to parthenogenesis may be common in simpler organisms but less likely as they become more complex ([21,43,46,64,65,83,86,87], but see [88] and citations herein). Some organisms have blocks to asexuality such as genomic imprinting in mammals [86] or paternally derived organelles in gymnosperms [21], which may represent actual ‘traps’ that impede asexual reproduction [89]. Livnat *et al*. [87] have proposed that selection for mixability and modularity promoted by sex from its origins should have had enormous influence in the architecture of sexual genomes, which may also have implications in the likelihood and success of an eventual shifting to asexual reproduction.

On the other hand, blocks to sexuality of asexual organisms may also be possible, and despite the fact that they may exist in obligate asexuals, they are rare in nature [35,63,83] perhaps because of the short longevity of pure-asexual lineages [25,37,83]. Evolutionary equilibria and cycles that result in our model show how sexual and asexual lineages may have successive opportunities to evolve blocking traits as side products of adaptive complexity [21,87], as well as the accumulation of other populational benefits and effects [12,13,19–23,25,30,47,90–92], which may help to explain how an evolving sexual lineage may persist up to accumulating complex differences without being replaced by asexual clones.

## Supplementary Material

R-script for running model simulations Function programed in R that allows generating the evolutionary dynamics of alleles determining (1) asexual (allele a in locus S) vs. sexual reproduction (allele s in locus S) and, in the second case, (2) non-costly sexual reproduction (allele n in locus M) vs. costly sexual reproduction (alle c in locus M).

## Data accessibility

Interested readers can explore the model and introduce all desired variations by using the R-script [61] in electronic supplementary material and at: https://github.com/JRubalcaba/Carranza-Polo.
