# Supplementary material for: Sexual reproduction with variable mating systems can resist asexuality in a rock–paper–scissors dynamics
Source: R Soc Open Sci. 2015 Jul 15;2(7):140383. doi: 10.1098/rsos.140383 (PMC4632567; doi:10.1098/rsos.140383)
Supplement: R-script for running model simulations Function programed in R that allows generating the evolutionary dynamics of alleles determining (1) asexual (allele a in locus S) vs. sexual reproduction (allele s in locus S) and, in the second case, (2) non-costly sexual reproduction (allele n in locus M) vs. [file rsos140383supp1.docx]

**Supplementary material to the paper:**

Sexual reproduction with variable mating systems can resist asexuality in a rock-paper-scissors dynamics

Juan Carranza and Vicente Polo

**R-script for running model simulations**

R-function programmed by

V. Polo, J.G. Rubalcaba and J. Carranza

**How to use**

The function programed in R allows generating the evolutionary dynamics of alleles determining (1) asexual (allele a in locus S) vs. sexual reproduction (allele s in locus S) and, in the second case, (2) non-costly sexual reproduction (allele n in locus M) vs. costly sexual reproduction (alle c in locus M).

**(1) Copy-paste and execute the function's code in R.**

**(2) Specify function parameters (see definition of parameters and example below) and store the output as an object:**

**Parameter Meaning**

m = Maximum potential number of mates for a polygynous male

R = Benefit of recombination with respect to asexuality

b = Proportion of male parental contribution accepted by nn female

k = Dominance effect of allele c in locus M

alpha = Probability of as individual choosing asexual strategy

a0 = Initial frequency of allele a (i.e., asexuality) in locus S

n0 = Initial frequency of allele n (i.e., non-costly sexuality) in locus M

gen = number of generations

**Output**

The function generates a matrix containing as many rows as generations (gen) and 9 columns. Each colum represent the dynamics of the variables necesary to estimate allele frequencies (see below). The last 4 columns are the allele frequencies of alleles a and s (locus S), n and c (locus M) respectively.

1 - pi: mean number of mates of homozygote nn males in the population

theta: mean number of mates of heterozygote cn males in the population

1 + phi: mean number of mates for polygynous cc males in the population

tao: proportion on sexual individuals in the population

a: allele at locus S influencing asexuality

s: allele at locus S influencing sexuality

n: allele at locus M influencing non-costly sex

c: allele at locus M influencing costly sex

output <- CarranzaandPolo(m = 3.3, R = 1.6, b = 0.5, k = 0.5, alpha = 0.5, a0 = 0.99, n0 = 0.99, gen = 10000)

**(3) Plot the dynamic of allele frequencies and the dynamic attractor using:**

plot(output$a, ylim = c(0,1), type = "l", col = "blue")

lines(output$c, col = "red")

plot(c ~ a, output, type = "l")

**RCode**

## The following function generates the dynamics of a rock-paper-scissors model that describe allele frequency dynamics of

# four alleles in two loci:

# (1) allele s determining sexual reproduction (locus S);

# (2) allele c determining costly sexual reproduction (e.g., polygynous breeding systems) (locus M);

# (3) allele a (= 1 - s) for asexual reproduction (locus S);

# (4) allele n (= 1 - c) for non-costly reproduction (locus M).

# Copy-paste the function's code in R and modify the parameters to explore the system dynamics

CarranzaandPolo <- function(data, m, R, b, k, alpha, a0, n0, gen) {

out <- list(data.frame())

for (i in 1 : length(R)) {

c0 <- 1 - n0

s0 <- 1 - a0

##Vectors 1

a <- numeric(gen)

s <- numeric(gen)

c <- numeric(gen)

n <- numeric(gen)

##Vectors 2

P1 <- numeric(gen)

P2 <- numeric(gen)

P3 <- numeric(gen)

P4 <- numeric(gen)

P5 <- numeric(gen)

P6 <- numeric(gen)

P7 <- numeric(gen)

P8 <- numeric(gen)

P9 <- numeric(gen)

##Vectors 3

denom <- numeric(gen)

pi <- numeric(gen)

tao <- numeric(gen)

phi <- numeric(gen)

theta <- numeric(gen)

##Vectors 4

x4x4 <- numeric(gen)

x4x5 <- numeric(gen)

x4x6 <- numeric(gen)

x4x7 <- numeric(gen)

x4x8 <- numeric(gen)

x4x9 <- numeric(gen)

x5x4 <- numeric(gen)

x5x5 <- numeric(gen)

x5x6 <- numeric(gen)

x5x7 <- numeric(gen)

x5x8 <- numeric(gen)

x5x9 <- numeric(gen)

x6x4 <- numeric(gen)

x6x5 <- numeric(gen)

x6x6 <- numeric(gen)

x6x7 <- numeric(gen)

x6x8 <- numeric(gen)

x6x9 <- numeric(gen)

x7x4 <- numeric(gen)

x7x5 <- numeric(gen)

x7x6 <- numeric(gen)

x7x7 <- numeric(gen)

x7x8 <- numeric(gen)

x7x9 <- numeric(gen)

x8x4 <- numeric(gen)

x8x5 <- numeric(gen)

x8x6 <- numeric(gen)

x8x7 <- numeric(gen)

x8x8 <- numeric(gen)

x8x9 <- numeric(gen)

x9x4 <- numeric(gen)

x9x5 <- numeric(gen)

x9x6 <- numeric(gen)

x9x7 <- numeric(gen)

x9x8 <- numeric(gen)

x9x9 <- numeric(gen)

##Vectors 5

sum1 <- numeric(gen)

sum2 <- numeric(gen)

sum3 <- numeric(gen)

sum4 <- numeric(gen)

sum5 <- numeric(gen)

sum6 <- numeric(gen)

sum7 <- numeric(gen)

sum8 <- numeric(gen)

sum9 <- numeric(gen)

sumT <- numeric(gen)

##Dynamics

for(i in 1 : gen) {

if (i > 1) {

P1[i] <- sum1[i - 1] / sumT[i - 1]

P2[i] <- sum2[i - 1] / sumT[i - 1]

P3[i] <- sum3[i - 1] / sumT[i - 1]

P4[i] <- sum4[i - 1] / sumT[i - 1]

P5[i] <- sum5[i - 1] / sumT[i - 1]

P6[i] <- sum6[i - 1] / sumT[i - 1]

P7[i] <- sum7[i - 1] / sumT[i - 1]

P8[i] <- sum8[i - 1] / sumT[i - 1]

P9[i] <- sum9[i - 1] / sumT[i - 1]

denom[i] <- 1 + (m - 1) * ((alpha * P6[i]) + P9[i] + k * ((alpha * P5[i]) + P8[i]))

pi[i] <- 1 / denom[i]

tao[i] <- (alpha * (P4[i] + P5[i] + P6[i])) + P7[i] + P8[i] + P9[i]

phi[i] <- (1 + (tao[i ] * (m - 1))) / denom[i ]

theta[i] <- (1 + tao[i ] * (k * (m - 1))) / denom[i ]

a[i] <- sum(P1[i ], P2[i ], P3[i ]) + 0.5 * sum(P4[i ], P5[i ], P6[i ])

c[i] <- P3[i ] + P6[i ] + P9[i ] + 0.5 * (P2[i ] + P5[i ] + P8[i ])

n[i] <- 1 - c[i ]

s[i] <- 1 - a[i ]

x4x4[i] <- alpha*alpha*P4[i]*P4[i]*R*pi[i]/tao[i]

x4x5[i] <- 0.5*alpha*alpha*pi[i]*P4[i]*P5[i]*R*(2-(k*(1-b)))/tao[i]

x4x6[i] <- 0.5*alpha*alpha*pi[i]*P4[i]*P6[i]*R*(1+b)/tao[i]

x4x7[i] <- alpha*pi[i]*P4[i]*P7[i]*R/tao[i]

x4x8[i] <- 0.5*alpha*pi[i]*P4[i]*P8[i]*R*(2-(k*(1-b)))/tao[i]

x4x9[i] <- 0.5*alpha*pi[i]*P4[i]*P9[i]*R*(1+b)/tao[i]

x5x4[i] <- 0.5*alpha*alpha*theta[i]*P4[i]*P5[i]*R*(2-k)/tao[i]

x5x5[i] <- 0.5*alpha*alpha*theta[i]*P5[i]*P5[i]*R*(2-k-(k*(1-k)*(1-b)))/tao[i]

x5x6[i] <- 0.5*alpha*alpha*theta[i]*P5[i]*P6[i]*R*(1+(b*(1-k)))/tao[i]

x5x7[i] <- 0.5*alpha*theta[i]*P5[i]*P7[i]*R*(2-k)/tao[i]

x5x8[i] <- 0.5*alpha*theta[i]*P5[i]*P8[i]*R*(2-k-(k*(1-k)*(1-b)))/tao[i]

x5x9[i] <- 0.5*alpha*theta[i]*P5[i]*P9[i]*R*(1+(b*(1-k)))/tao[i]

x6x4[i] <- 0.5*alpha*alpha*P4[i]*P6[i]*R*phi[i]/tao[i]

x6x5[i] <- 0.5*alpha*alpha*P5[i]*P6[i]*phi[i]*R/tao[i]

x6x6[i] <- 0.5*alpha*alpha*phi[i]*P6[i]*P6[i]*R/tao[i]

x6x7[i] <- 0.5*alpha*phi[i]*R*P6[i]*P7[i]/tao[i]

x6x8[i] <- 0.5*alpha*phi[i]*R*P6[i]*P8[i]/tao[i]

x6x9[i] <- 0.5*alpha*R*phi[i]*P6[i]*P9[i]/tao[i]

x7x4[i] <- alpha*pi[i]*R*P4[i]*P7[i]/tao[i]

x7x5[i] <- 0.5*alpha*R*pi[i]*P5[i]*P7[i]*(2-(k*(1-b)))/tao[i]

x7x6[i] <- 0.5*alpha*pi[i]*R*P6[i]*P7[i]*(1+b)/tao[i]

x7x7[i] <- R*pi[i]*P7[i]*P7[i]/tao[i]

x7x8[i] <- 0.5*R*pi[i]*P7[i]*P8[i]*(2-(k*(1-b)))/tao[i]

x7x9[i] <- 0.5*R*pi[i]*P7[i]*P9[i]*(1+b)/tao[i]

x8x4[i] <- 0.5*alpha*theta[i]*R*P4[i]*P8[i]*(2-k)/tao[i]

x8x5[i] <- 0.5*alpha*R*theta[i]*P5[i]*P8[i]*(2-k-(k*(1-k)*(1-b)))/tao[i]

x8x6[i] <- 0.5*alpha*theta[i]*R*P6[i]*P8[i]*(1+(b*(1-k)))/tao[i]

x8x7[i] <- 0.5*theta[i]*R*P7[i]*P8[i]*(2-k)/tao[i]

x8x8[i] <- 0.5*theta[i]*R*P8[i]*P8[i]*(2-k-(k*(1-k)*(1-b)))/tao[i]

x8x9[i] <- 0.5*theta[i]*R*P8[i]*P9[i]*(1+(b*(1-k)))/tao[i]

x9x4[i] <- 0.5*alpha*R*phi[i]*P4[i]*P9[i]/tao[i]

x9x5[i] <- 0.5*alpha*R*phi[i]*P5[i]*P9[i]/tao[i]

x9x6[i] <- 0.5*alpha*R*phi[i]*P6[i]*P9[i]/tao[i]

x9x7[i] <- 0.5*R*phi[i]*P7[i]*P9[i]/tao[i]

x9x8[i] <- 0.5*R*phi[i]*P8[i]*P9[i]/tao[i]

x9x9[i] <- 0.5*R*phi[i]*P9[i]*P9[i]/tao[i]

sum1[i] <- P1[i] + 0.25 * x4x4[i] + 0.175 * x4x5[i] + 0.175 * x5x4[i] + 0.0875 * x5x5[i]

sum2[i] <- P2[i] + 0.175 * x4x5[i] + 0.25 * x4x6[i] + 0.175 * (x5x4[i] + x5x5[i] + x5x6[i]) + 0.25 * x6x4[i] + 0.175 * x6x5[i]

sum3[i] <- P3[i] + 0.0875 * x5x5[i] + 0.175 * x5x6[i] + 0.175 * x6x5[i] + 0.25 * x6x6[i]

sum4[i] <- ((1-alpha)*P4[i])+0.5*x4x4[i]+0.25*x4x5[i]+0.5*x4x7[i]+0.25*x4x8[i]+0.25*x5x4[i]+0.175*x5x5[i]+0.25*x5x7[i]+0.175*x5x8[i]+0.5*x7x4[i]+0.25*x7x5[i]+0.25*x8x4[i]+0.175*x8x5[i]

sum5[i] <- ((1-alpha)*P5[i])+0.25*x4x5[i]+0.5*x4x6[i]+0.25*x4x8[i]+0.5*x4x9[i]+0.25*(x5x4[i]+x5x5[i]+x5x6[i]+x5x7[i]+x5x8[i]+x5x9[i])+0.5*x6x4[i]+0.25*x6x5[i]+0.5*x6x7[i]+0.25*x6x8[i]+0.25*x7x5[i]+0.5*x7x6[i]+0.25*(x8x4[i]+x8x5[i]+x8x6[i])+0.5*x9x4[i]+0.25*x9x5[i]

sum6[i] <- ((1-alpha)*P6[i])+0.175*x5x5[i]+0.25*x5x6[i]+0.175*x5x8[i]+0.25*x5x9[i]+0.25*x6x5[i]+0.5*x6x6[i]+0.25*x6x8[i]+0.5*x6x9[i]+0.175*x8x5[i]+0.25*x8x6[i]+0.25*x9x5[i]+0.5*x9x6[i]

sum7[i] <- 0.25*x4x4[i]+0.175*x4x5[i]+0.5*x4x7[i]+0.25*x4x8[i]+0.175*x5x4[i]+0.0875*x5x5[i]+0.25*x5x7[i]+0.175*x5x8[i]+0.5*x7x4[i]+0.25*x7x5[i]+x7x7[i]+0.5*x7x8[i]+0.25*x8x4[i]+0.175*x8x5[i]+0.5*x8x7[i]+0.25*x8x8[i]

sum8[i] <- 0.175*x4x5[i]+0.25*(x4x6[i]+x4x8[i])+0.5*x4x9[i]+0.175*(x5x4[i]+x5x5[i]+x5x6[i])+0.25*(x5x7[i]+x5x8[i]+x5x9[i])+0.25*x6x4[i]+0.175*x6x5[i]+0.5*x6x7[i]+0.25*x6x8[i]+0.25*x7x5[i]+0.5*(x7x6[i]+x7x8[i])+x7x9[i]+0.25*(x8x4[i]+x8x5[i]+x8x6[i])+0.5*(x8x7[i]+x8x8[i]+x8x9[i])+0.5*x9x4[i]+0.25*x9x5[i]+x9x7[i]+0.5*x9x8[i]

sum9[i] <- 0.0875*x5x5[i]+0.175*(x5x6[i]+x5x8[i])+0.25*x5x9[i]+0.175*x6x5[i]+0.25*(x6x6[i]+x6x8[i])+0.5*x6x9[i]+0.175*x8x5[i]+0.25*(x8x6[i]+x8x8[i])+0.5*x8x9[i]+0.25*x9x5[i]+0.5*(x9x6[i]+x9x8[i])+x9x9[i]

sumT[i] <- sum(sum1[i ], sum2[i ], sum3[i ], sum4[i ], sum5[i ], sum6[i ], sum7[i ], sum8[i ], sum9[i ])

}else{

a[1] <- a0

s[1] <- s0

c[1] <- c0

n[1] <- n0

P1[i] <- a[i]^2 * n[i]^2

P2[i] <- 2 * a[i]^2 * n[i] * c[i]

P3[i] <- a[i]^2 * c[i]^2

P4[i] <- 2 * a[i] * s[i] * n[i]^2

P5[i] <- 4 * a[i] * s[i] * n[i] * c[i]

P6[i] <- 2 * a[i] * s[i] * c[i]^2

P7[i] <- s[i]^2 * n[i]^2

P8[i] <- 2 * s[i]^2 * n[i] * c[i]

P9[i] <- s[i]^2 * c[i]^2

denom[i] <- 1 + (m - 1) * ((alpha * P6[i]) + P9[i] + k * ((alpha[i] * P5[i]) + P8[i]))

pi[i] <- 1 / denom[i]

tao[i] <- (alpha * (P4[i] + P5[i] + P6[i])) + P7[i] + P8[i] + P9[i]

phi[i] <- (1 + (tao[i] * (m - 1))) / denom[i]

theta[i] <- (1 + tao[i] * (k * (m-1))) / denom[i]

x4x4[i] <- alpha*alpha*P4[i]*P4[i]*R*pi[i]/tao[i]

x4x5[i] <- 0.5*alpha*alpha*pi[i]*P4[i]*P5[i]*R*(2-(k*(1-b)))/tao[i]

x4x6[i] <- 0.5*alpha*alpha*pi[i]*P4[i]*P6[i]*R*(1+b)/tao[i]

x4x7[i] <- alpha*pi[i]*P4[i]*P7[i]*R/tao[i]

x4x8[i] <- 0.5*alpha*pi[i]*P4[i]*P8[i]*R*(2-(k*(1-b)))/tao[i]

x4x9[i] <- 0.5*alpha*pi[i]*P4[i]*P9[i]*R*(1+b)/tao[i]

x5x4[i] <- 0.5*alpha*alpha*theta[i]*P4[i]*P5[i]*R*(2-k)/tao[i]

x5x5[i] <- 0.5*alpha*alpha*theta[i]*P5[i]*P5[i]*R*(2-k-(k*(1-k)*(1-b)))/tao[i]

x5x6[i] <- 0.5*alpha*alpha*theta[i]*P5[i]*P6[i]*R*(1+(b*(1-k)))/tao[i]

x5x7[i] <- 0.5*alpha*theta[i]*P5[i]*P7[i]*R*(2-k)/tao[i]

x5x8[i] <- 0.5*alpha*theta[i]*P5[i]*P8[i]*R*(2-k-(k*(1-k)*(1-b)))/tao[i]

x5x9[i] <- 0.5*alpha*theta[i]*P5[i]*P9[i]*R*(1+(b*(1-k)))/tao[i]

x6x4[i] <- 0.5*alpha*alpha*P4[i]*P6[i]*R*phi[i]/tao[i]

x6x5[i] <- 0.5*alpha*alpha*P5[i]*P6[i]*phi[i]*R/tao[i]

x6x6[i] <- 0.5*alpha*alpha*phi[i]*P6[i]*P6[i]*R/tao[i]

x6x7[i] <- 0.5*alpha*phi[i]*R*P6[i]*P7[i]/tao[i]

x6x8[i] <- 0.5*alpha*phi[i]*R*P6[i]*P8[i]/tao[i]

x6x9[i] <- 0.5*alpha*R*phi[i]*P6[i]*P9[i]/tao[i]

x7x4[i] <- alpha*pi[i]*R*P4[i]*P7[i]/tao[i]

x7x5[i] <- 0.5*alpha*R*pi[i]*P5[i]*P7[i]*(2-(k*(1-b)))/tao[i]

x7x6[i] <- 0.5*alpha*pi[i]*R*P6[i]*P7[i]*(1+b)/tao[i]

x7x7[i] <- R*pi[i]*P7[i]*P7[i]/tao[i]

x7x8[i] <- 0.5*R*pi[i]*P7[i]*P8[i]*(2-(k*(1-b)))/tao[i]

x7x9[i] <- 0.5*R*pi[i]*P7[i]*P9[i]*(1+b)/tao[i]

x8x4[i] <- 0.5*alpha*theta[i]*R*P4[i]*P8[i]*(2-k)/tao[i]

x8x5[i] <- 0.5*alpha*R*theta[i]*P5[i]*P8[i]*(2-k-(k*(1-k)*(1-b)))/tao[i]

x8x6[i] <- 0.5*alpha*theta[i]*R*P6[i]*P8[i]*(1+(b*(1-k)))/tao[i]

x8x7[i] <- 0.5*theta[i]*R*P7[i]*P8[i]*(2-k)/tao[i]

x8x8[i] <- 0.5*theta[i]*R*P8[i]*P8[i]*(2-k-(k*(1-k)*(1-b)))/tao[i]

x8x9[i] <- 0.5*theta[i]*R*P8[i]*P9[i]*(1+(b*(1-k)))/tao[i]

x9x4[i] <- 0.5*alpha*R*phi[i]*P4[i]*P9[i]/tao[i]

x9x5[i] <- 0.5*alpha*R*phi[i]*P5[i]*P9[i]/tao[i]

x9x6[i] <- 0.5*alpha*R*phi[i]*P6[i]*P9[i]/tao[i]

x9x7[i] <- 0.5*R*phi[i]*P7[i]*P9[i]/tao[i]

x9x8[i] <- 0.5*R*phi[i]*P8[i]*P9[i]/tao[i]

x9x9[i] <- 0.5*R*phi[i]*P9[i]*P9[i]/tao[i]

sum1[i] <- P1[i] + 0.25 * x4x4[i] + 0.175 * x4x5[i] + 0.175 * x5x4[i] + 0.0875 * x5x5[i]

sum2[i] <- P2[i] + 0.175 * x4x5[i] + 0.25 * x4x6[i] + 0.175 * (x5x4[i] + x5x5[i] + x5x6[i]) + 0.25 * x6x4[i] + 0.175 * x6x5[i]

sum3[i] <- P3[i] + 0.0875 * x5x5[i] + 0.175 * x5x6[i] + 0.175 * x6x5[i] + 0.25 * x6x6[i]

sum4[i] <- ((1-alpha)*P4[i])+0.5*x4x4[i]+0.25*x4x5[i]+0.5*x4x7[i]+0.25*x4x8[i]+0.25*x5x4[i]+0.175*x5x5[i]+0.25*x5x7[i]+0.175*x5x8[i]+0.5*x7x4[i]+0.25*x7x5[i]+0.25*x8x4[i]+0.175*x8x5[i]

sum5[i] <- ((1-alpha)*P5[i])+0.25*x4x5[i]+0.5*x4x6[i]+0.25*x4x8[i]+0.5*x4x9[i]+0.25*(x5x4[i]+x5x5[i]+x5x6[i]+x5x7[i]+x5x8[i]+x5x9[i])+0.5*x6x4[i]+0.25*x6x5[i]+0.5*x6x7[i]+0.25*x6x8[i]+0.25*x7x5[i]+0.5*x7x6[i]+0.25*(x8x4[i]+x8x5[i]+x8x6[i])+0.5*x9x4[i]+0.25*x9x5[i]

sum6[i] <- ((1-alpha)*P6[i])+0.175*x5x5[i]+0.25*x5x6[i]+0.175*x5x8[i]+0.25*x5x9[i]+0.25*x6x5[i]+0.5*x6x6[i]+0.25*x6x8[i]+0.5*x6x9[i]+0.175*x8x5[i]+0.25*x8x6[i]+0.25*x9x5[i]+0.5*x9x6[i]

sum7[i] <- 0.25*x4x4[i]+0.175*x4x5[i]+0.5*x4x7[i]+0.25*x4x8[i]+0.175*x5x4[i]+0.0875*x5x5[i]+0.25*x5x7[i]+0.175*x5x8[i]+0.5*x7x4[i]+0.25*x7x5[i]+x7x7[i]+0.5*x7x8[i]+0.25*x8x4[i]+0.175*x8x5[i]+0.5*x8x7[i]+0.25*x8x8[i]

sum8[i] <- 0.175*x4x5[i]+0.25*(x4x6[i]+x4x8[i])+0.5*x4x9[i]+0.175*(x5x4[i]+x5x5[i]+x5x6[i])+0.25*(x5x7[i]+x5x8[i]+x5x9[i])+0.25*x6x4[i]+0.175*x6x5[i]+0.5*x6x7[i]+0.25*x6x8[i]+0.25*x7x5[i]+0.5*(x7x6[i]+x7x8[i])+x7x9[i]+0.25*(x8x4[i]+x8x5[i]+x8x6[i])+0.5*(x8x7[i]+x8x8[i]+x8x9[i])+0.5*x9x4[i]+0.25*x9x5[i]+x9x7[i]+0.5*x9x8[i]

sum9[i] <- 0.0875*x5x5[i]+0.175*(x5x6[i]+x5x8[i])+0.25*x5x9[i]+0.175*x6x5[i]+0.25*(x6x6[i]+x6x8[i])+0.5*x6x9[i]+0.175*x8x5[i]+0.25*(x8x6[i]+x8x8[i])+0.5*x8x9[i]+0.25*x9x5[i]+0.5*(x9x6[i]+x9x8[i])+x9x9[i]

sumT[i] <- sum(sum1[i ], sum2[i ], sum3[i ], sum4[i ], sum5[i ], sum6[i ], sum7[i ], sum8[i ], sum9[i ])

}

}

#Output

output <- as.data.frame(cbind(pi, theta, phi, tao, denom, a, s, n, c))

names(output) <- c("1 - pi", "theta", "1 + phi", "tao", "denom", "a", "s", "n", "c")

return(output)

}

}

# Recomended parameters and plotting:

output <- CarranzaandPolo(m = 3.3, R = 1.6, b = 0.5, k = 0.5, alpha = 0.5, a0 = 0.99, n0 = 0.99, gen = 10000)

#Plot the dynamic of allele requency asexual reproduction vs. costly sexual reproduction (a vs. c):

plot(output$a, ylim = c(0,1), type = "l", col = "blue")

lines(output$c, col = "red")

#Plot the dynamic attractor

plot(c ~ a, output, type = "l")
